# Sustainable Reinforcement of Silicone Rubber: Comparative Analysis of Biosilica from Rice Husk and Conventional Silica

**DOI:** 10.3390/polym17030406

**Published:** 2025-02-03

**Authors:** Hyeon Woo Jeong, Kyoung Tae Park, Su Min Oh, Sang Eun Shim, Yingjie Qian

**Affiliations:** 1 Guangzhou Institute of Energy Conversion, Chinese Academy of Sciences, NengYuan Street 2, Tianhe District, Guangzhou 510640, China; 2 Department of Chemistry and Chemical Engineering, Education and Research Center for Smart Energy and Materials, Inha University, Incheon 22212, Republic of Korea

**Keywords:** silicone rubber, rice husk, biosilica, Payne effect

## Abstract

The objective of this study is to compare rice husk-derived silica (biosilica) synthesized via an environmentally friendly method with conventional silica (Zeosil 175) for reinforcing the mechanical properties of silicone rubber. The silanol group content of Zeosil 175 (9.45 OH/nm^2^) is higher than that of biosilica (7.07 OH/nm^2^), whereas the specific surface area of biosilica (159.52 m^2^/g) exceeds that of Zeosil 175 (144.90 m^2^/g). Silicone rubber specimens containing two types of silica nanoparticles were prepared at loading levels of 5, 10, 15, 20, 25, and 30 parts per hundred rubber to evaluate their mechanical properties and characteristics. Results indicate that silicone rubber filled with biosilica shows comparable tensile strength to Zeosil 175 at low filler contents, which can be attributed to its higher specific surface area. However, at higher loading levels, the mechanical properties are somewhat diminished due to the Payne effect and filler agglomeration resulting from the larger particle size of biosilica. These experimental findings offer insights into the potential utilization of rice husk-derived biosilica as an alternative to conventional silica in enhancing the properties of silicone rubber alongside the findings of the mechanical analysis.

## 1. Introduction

In today’s highly developed world, environmental issues have emerged as a significant global concern, presenting challenges that are difficult to overcome. Advancements in agricultural technology and increased productivity have resulted in higher rice production, generating rice husk as an abundant byproduct that contributes to environmental waste [1,2]. One potential solution is to utilize rice husk as an energy resource or recycle it for reuse. Composed primarily of organic materials such as hemicellulose, cellulose, and lignin, rice husk contains approximately 10 to 20% residual ash [3,4]. Upon combustion, rice husk produces rice husk ash (RHA), which consists of more than 83 to 90% silica [4]. Various studies have successfully extracted pure silica from rice husk ash through processes such as titration and precipitation, achieving purity levels exceeding 98% and thereby reducing the environmental impact of rice husk [4,5,6,7,8,9]. Silicone elastomers have become essential in various fields, including construction, aerospace, electronics, biomedicine, and artificial intelligence, due to their exceptional insulation, durability, low surface energy, and compatibility with biological systems [10,11,12,13,14]. In contrast to conventional rubber, which has a C–C backbone, silicone rubber exhibits superior weatherability, thermal stability, chemical stability, and low toxicity [15]. However, silicone rubber displays inferior mechanical properties, such as tensile strength and tear strength, compared to conventional organic rubbers [16]. Numerous researchers have explored methods to mitigate this issue by incorporating fillers, including carbon black and other inorganic materials. Silica is a representative inorganic filler to reinforce the mechanical properties of rubber. In silicone rubber, silica especially reinforces strengths not only through physical filler–matrix interaction but also through hydrogen bonding between the silanol groups on the surface of silica and the oxygen atoms in the backbone chain of silicone rubber [17].

Many previous studies have demonstrated the reinforcement of rubber properties by incorporating silica. Among these, research by Boonstra et al. demonstrated the significant role of silica-silicone rubber interactions in enhancing mechanical properties. This study showed that strong hydrogen bonding forms between the silica particles and the silicone rubber matrix, as well as among the filler particles themselves, resulting in substantial reinforcement [18]. Additionally, many studies have examined biosilica derived from rice husk and other bio-resources, applying it across various composites. For instance, Gomes et al. explored the use of rice husk silica in magnesium-based Sorel cements, demonstrating its effectiveness in improving water resistance and mechanical properties by reducing soluble phases and enhancing magnesium silicate hydrate (M-S-H) formation [19]. Eissa et al. investigated ethylene propylene diene monomer (EPDM)/nitrile butadiene rubber (NBR) composites incorporating rice husk silica (RHS) and other fillers, demonstrating that RHS effectively enhanced thermal stability and swelling resistance in motor oil, highlighting its potential as a supporting filler for carbon black-reinforced rubber vulcanizates [20]. Choophun et al. explored the effect of silica derived from rice husk on the mechanical properties of natural rubber composites, showing that silica loading enhances tensile strength, hardness, and modulus, while reducing elongation and abrasion resistance with optimal performance observed at 20 phr silica content [21].

Furthermore, Sethuramalingam et al. explored Nitrile butyl rubber composites reinforced with silica and RHA, demonstrating that filler loading enhanced tensile strength and hardness, with optimal tear strength observed at 20 phr for silica-RHA blends [22]. Jiang et al. examined the properties of polydimethylsiloxane silicone rubber by incorporating both precipitated and fumed silica, analyzing their effects on physical, electrical, and surface properties [23]. Azmi et al. investigated the use of silica, derived from both commercial sources (CS) and RHA, as fillers in polydimethylsiloxane (PDMS) composites for vibration absorption. They found that incorporating SiO_2_ at 2–6 wt% improved the vibration-damping properties of PDMS, with the PDMS-RHA composite exhibiting superior vibration reduction compared to the PDMS-CS composite [24]. However, to the best of our knowledge, studies investigating the mechanical properties of silicone rubber with varying loadings of biosilica and commercial silica have yet to be conducted.

This study analyzed and compared biosilica derived from rice husk and conventional silica (Zeosil 175) to evaluate their potential for reinforcing silicone rubber. The investigation assessed how hydroxyl group content, specific surface area, and particle size influenced mechanical properties at various filler loadings. Additionally, the role of the Payne effect in determining filler dispersion and its implications for material performance was evaluated, providing insights into the application of biosilica as a sustainable reinforcing agent for silicone rubber.

## 2. Materials and Methods

### 2.1. Materials

The rice husk used for biosilica production was sourced from a rice warehouse in Yeongju (Republic of Korea). Precipitated silica (Zeosil 175), used for comparison with biosilica, was obtained from Solvay Korea (Seoul, Republic of Korea). Hydrochloric acid (HCl, 35% *v*/*v*) and sodium hydroxide (NaOH) were purchased from Sigma-Aldrich Korea (Seoul, Republic of Korea). The silicone gum employed for manufacturing the silicone rubber sheet was T 722TM (vinyl content: 0.03 mol%, molecular weight: 600,000 g/mol; vinyl-terminated PDMS), procured from Grace Continental Korea Co., Ltd. (Bucheon, Republic of Korea). A peroxide curative was prepared by mixing 2,5-dimethyl-2,5-di(tert-butylperoxy) hexane with vinyl silicone gum in a 1:1 ratio; this mixture was obtained from Grace Continental Korea Co., Ltd. (Bucheon, Republic of Korea). All reagents were used without further purification.

### 2.2. Preparation of Bio-Generated Silica (Biosilica)

**Figure 1:**
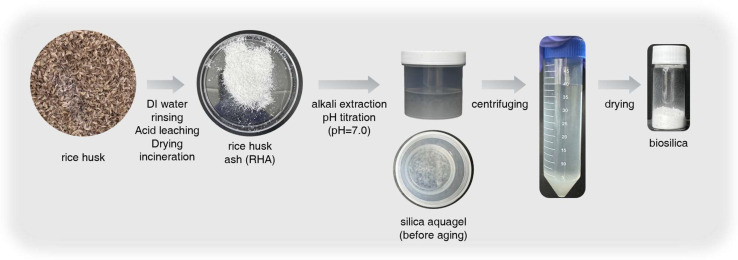
Schematic of extraction biosilica process from rice husk ash by alkali extraction and titration.

The method for extracting high-purity silica from rice husks has been extensively studied by researchers, with particular reference to the works of Kalapathy et al. and Liou et al. [8,25]. The procedure for synthesizing biosilica is illustrated in Figure 1. Rice husks were rinsed with deionized (DI) water and dried at 100 °C overnight. The rinsed rice husks were then treated with a 3 M hydrochloric acid (HCl) solution at 100 °C for 1 h to remove metallic impurities prior to silica extraction. The mixture was then filtered using a glass filter and dried at 110 °C for 24 h. For the combustion process, the dried rice husks were placed in a ceramic combustion boat and subjected to a muffle furnace at 700 °C, with a heating rate of 5 °C/min for 2 h in an air atmosphere. The rice husk ash (RHA) was subsequently stirred in a 1 M sodium hydroxide (NaOH) solution (RHA:NaOH solution = 1:10, *v*/*v*) at 80 °C for 1 h. The resulting solution was centrifuged to remove carbon solids and filtered through a glass filter (8–12 µm) until a clear and colorless solution was obtained, which was then diluted to 1.0 M. Next, a 1.0 M HCl solution was gradually added with constant agitation, raising the pH to 7 over a 30 min reaction period, resulting in the precipitation of Aquagel. DI Water was then added, and the mixture was centrifuged and dried at 80 °C for 24 h in a vacuum chamber to prevent changes in surface area and morphology due to agglomeration. Finally, the dried material was crushed using an agate mortar to obtain biosilica grains, which were then sieved through a 200-mesh sieve. The *yield of biosilica* was calculated through the equation below:

(1) $$Yield\;of\;biosilica\;(wt\%)=\frac{Weight\;of\;biosilica\;obtained}{Weight\;of\;silica\;in\;RHA}\times 100\%$$

### 2.3. Fabrication of Silicone Rubber Sheets Filled with Silica

Table 1 summarizes the formulation and composition of silicone rubber composites. The composites were prepared by incorporating either biosilica or Zeosil 175 into the silicone rubber matrix at various loading levels. The specimens were designated as BSi x and PSi x for biosilica and Zeosil 175-containing composites, respectively, where x denotes the filler loading in parts per hundred rubber (phr). The filler content varied from 0 phr (pristine silicone rubber used as a control) to 30 phr, in increments of 5 phr.

The composite preparation began with processing 100 phr of T 722TM using a two-roll mill (BST-T6, Bongshin Tech. Ltd., Incheon, Republic of Korea) mixer. The silica filler was gradually incorporated into the matrix in 5 phr increments at 3 min intervals. Following the complete addition of the prescribed silica amount, the mixture was further milled for 5 min to ensure homogeneous filler dispersion. Subsequently, 1 phr of 2,5-dimethyl-2,5-di(tert-butylperoxy) hexane was introduced as a vulcanization catalyst, followed by an additional 5 min of milling.

For the vulcanization process, 50 g of each composite formulation was precisely weighed and centrally positioned in a mold (150 mm × 150 mm × 2 mm). Vulcanization was performed in two stages: initial vulcanization through compression molding at 170 °C for 10 min, a curing time selected to ensure complete cross-linking while avoiding material degradation, followed by post-curing in a forced-air circulation oven at 200 °C for 4 h.

### 2.4. Characterizations of Silica Nanoparticles

#### 2.4.1. X-Ray Diffraction (XRD)

To compare the crystallinity of biosilica and conventional precipitated silica (Zeosil 175), powder X-ray diffraction patterns were recorded at ambient temperature on a PANalytical X’pert Pro MRD diffractometer (Warszawa, Poland) using CuKα radiation (λ = 1.5406 Å) at 40 kV and 30 mA. The data were collected in a θ-2θ scan mode from 10° to 90° with a step size of 0.02° and a scan speed of 3°/min.

#### 2.4.2. Attenuated Total Reflection Fourier Transform Infrared Spectroscopy (ATR-FTIR)

Attenuated total reflection Fourier transform infrared spectroscopy spectra were recorded using a Spectrum 2 spectrometer (PerkinElmer, Waltham, MA, USA). Measurements were conducted with a resolution of 1 cm^−1^ over 16 scans, covering the spectral range of 4000 to 600 cm^−1^. Silica nanoparticles were dried overnight in a vacuum oven to remove residual moisture.

#### 2.4.3. Scanning Electron Microscopy (SEM)

The morphology of biosilica and Zeosil 175 and the dispersion of each silica nanoparticle in silicone rubber was examined by scanning electron microscopy using HITACHI SU 8010 (Tokyo, Japan) at an accelerating voltage of 15 kV. Samples were sputter-coated with platinum to enhance conductivity prior to SEM imaging.

#### 2.4.4. Specific Surface Area

The specific surface area and total pore volume of biosilica and Zeosil 175 were estimated from N_2_ adsorption–desorption isotherm measured with BELSOLP-MAX II (Osaka, Japan) at 77 K before the measurement; each silica was pretreated in a vacuum at 120 °C for 24 h.

#### 2.4.5. Thermogravimetric Analysis (TGA)

Thermogravimetric analysis was performed using a TGA 4000 analyzer (PerkinElmer, Waltham, MA, USA). SiO_2_ nanoparticles were dried in a vacuum oven overnight to remove water. A specific method was employed to quantify the silanol groups on the surface of silica [26,27]. Samples were heated in a nitrogen atmosphere from 30 °C to 120 °C at 10 °C/min and held at this temperature for 10 min and heated at 20 °C/min to 800 °C and held for 10 min under nitrogen atmosphere.

#### 2.4.6. X-Ray Fluorescence (XRF)

To compare the composition and purity of each two types of silica, the chemical composition of biosilica and Zeosil 175 was measured using X-ray fluorescence (XRF) using ZSX Primus IV (Rigaku, Osaka, Japan).

### 2.5. Characterizations of Silicone Rubber

#### 2.5.1. ATR-FTIR

ATR-FTIR spectra were recorded using a Spectrum 2 spectrometer (PerkinElmer, Waltham, MA, USA). Measurements were conducted with a resolution of 1 cm^−1^ over 16 scans, covering the spectral range of 4000 to 600 cm^−1^.

#### 2.5.2. TGA

Thermogravimetric analysis was performed using a TGA 4000 analyzer (PerkinElmer, Waltham, MA, USA). During the silicone rubber characterization process, samples were heated from 30 °C to 800 °C at a ramping rate of 10 °C/min under a nitrogen atmosphere. The weight loss rate curves for all samples were obtained by differentiating the TGA thermograms.

#### 2.5.3. SEM

The morphology of biosilica and Zeosil 175 and the dispersion of each silica nanoparticle in silicone rubber were examined by scanning electron microscopy using HITACHI SU 8010 (Tokyo, Japan) at an accelerating voltage of 15 kV. Before characterization, silicone rubber specimens were cryogenically frozen in liquid nitrogen and fractured, and the morphology of the fracture surfaces was analyzed. Samples were sputter-coated with platinum to enhance conductivity prior to SEM imaging.

#### 2.5.4. Tensile and Tear Properties

Tensile and tear properties were examined using a Universal Test Machine (UTM, DUI-1TCM, Daekyung Engineering Co., Ltd., Bucheon, Republic of Korea) with a load cell of 1 kN and crosshead speed of 500 mm/min. Specimens of tensile and tear tests were prepared according to ASTM D412 [28] and ASTM D624 [29], respectively. Dumbbell and crescent specimens were cut from a vulcanized silicone rubber sheet with a thickness of 2 mm.

#### 2.5.5. Crosslinking Density

The crosslinking density of vulcanized silicone rubber was quantitatively determined via the solvent-swelling method using Soxhlet extraction with toluene as the extracting solvent. The extraction process was conducted for a duration of 24 h. The swollen samples were weighed immediately after the solvent was removed. Then, samples were dried in a vacuum oven at 50 °C for 24 h, and the weights of dried samples were measured. The crosslinking density was calculated using the Flory–Rehner equation [30].

(2) ve=−[ln⁡1−Vr+Vr+ΧVr2]V1Vr13−12Vr

where $v_{e}$ is the number of crosslinks per unit volume, $V_{1}$ is the solvent molar volume, which is 106.2 cm^3^/mol for toluene, and $V_{r}$ is the gel volume in the swollen sample. $V_{r}$ was obtained through the equation below.

(3) Vr=mrρrmrρr+msρs

$m_{r}$ is the mass of the dried sample, and $\rho_{r}$ is the density of the dried sample, $m_{s}$ is the weight of the solvent absorbed by the sample, and $\rho_{s}$ is the solvent density, which is 0.867 g/cm^3^ for toluene.

Χ is the polymer–solvent interaction parameter which was calculated by the equation below [31,32].

(4) $$Χ=\frac{V_{1}}{RT}{\left(\delta_{r}-\delta_{s}\right)}^{2}$$

where $\delta_{r}$ is the PDMS solubility parameter (7.3 (cal/cm^3^)^1/2^), $\delta_{s}$ is the toluene solubility parameter (8.9 (cal/cm^3^)^1/2^), *T* is the absolute temperature (K), and *R* is the gas constant (1.987 cal/mol∙K). The value of Χ calculated using these values is 0.46, which is in good agreement with the values in the reference [33,34].

#### 2.5.6. Dynamic Mechanical Properties of Uncured Silicone Compound and Cured Silicone Rubber

The analysis of viscoelastic properties and curing characteristics of uncured silicone compounds and cured silicone rubber was determined by Rubber Process Analyzer (RPA, RPA-V1, U-Can Dynatex Inc., Taichung, Taiwan). The maximum and minimum torque, scorch time (ts2), and optimum cure time (tc90) of silicone rubber filled with each type of silica were determined by analyzing the curing characteristics at 170 °C for 10 min, with a frequency of 1.67 Hz and an oscillation amplitude of 1%. Dynamic rheological tests of uncured silicone compounds were performed using an amplitude sweep at a constant frequency of 1 Hz, with an oscillation strain range from 0.1% to 400% at 60 °C.

## 3. Results and Discussion

### 3.1. Structures and Properties of Biosilica and Zeosil 175

#### 3.1.1. XRD of Silica

**Figure 2:**
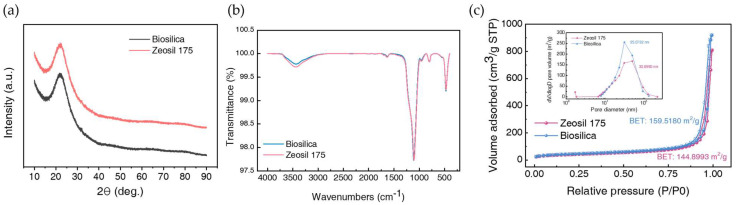
(**a**) X-ray diffraction (XRD) patterns of Biosilica and Zeosil 175. (**b**) FT-IR of biosilica and Zeosil 175. (**c**) Nitrogen adsorption–desorption isotherms and corresponding Barrett–Joyner–Halenda (BJH) pore size distribution (inset) of Zeosil 175 and biosilica.

The X-ray diffraction patterns of biosilica and Zeosil 175 are presented in Figure 2a. Both silica fillers exhibit similar peak profiles, with a prominent broad peak at 2θ = 22.5°, corresponding to their Full Width at Half-Maximum (FWHM). The broad diffraction peaks indicate that the silica fillers lack long-range crystalline order and possess a predominantly disordered structure. This amorphous characteristic is a common feature of synthetic silica materials produced through various manufacturing processes [35,36].

#### 3.1.2. FT-IR of Silica

The FT-IR spectra of biosilica and Zeosil 175, presented in Figure 2b, exhibit similar transmission characteristics. A broad transmission peak in the range of 3200 to 3600 cm^−1^, along with a peak near 1650 cm^−1^, corresponds to O–H stretching and bending vibrations, respectively [37]. These peaks indicate the presence of abundant silanol groups on the surface of nanosilica. Additionally, the peak at nearly 1100 cm^−1^ and 800 cm^−1^ means Si-O-Si stretching bond in nanosilica [37]. The FT-IR spectra allow for an indirect comparison of the silanol group content on the surface of each silica type. The intensity of the peak for Zeosil 175 was slightly stronger than that of biosilica, suggesting a higher OH group content on the surface of Zeosil 175, which could lead to stronger hydrogen bonding between the silica surface and the matrix. However, this increased content may also result in stronger filler–filler interactions, potentially reinforcing the rubber while also promoting agglomeration.

#### 3.1.3. BET of Silica

The nitrogen adsorption–desorption isotherms and pore size distributions, illustrated in Figure 2c, demonstrate the differences in specific surface area and pore characteristics between biosilica and Zeosil 175. This analysis indicates that both silicas are not microporous or macroporous but rather mesoporous [38]. The specific surface area of biosilica is larger (159.52 m^2^/g) than that of Zeosil 175 (144.90 m^2^/g). In terms of specific surface area, biosilica is more advantageous for forming hydrogen bonds with the matrix or other fillers compared to Zeosil 175. The average pore diameters of biosilica and Zeosil 175 are 35.07 and 30.90 nm, respectively. Although smaller pore sizes generally provide a larger exposed surface area conducive to interactions with matrices or other fillers, the graph’s profile reveals that the pore size distribution is not narrow, and Zeosil 175 exhibits larger pore diameters exceeding 100 nm. Thus, inferring the extent of interactions solely based on pore diameter presents considerable analytical challenges.

#### 3.1.4. The Morphology of Biosilica and Zeosil 175

**Figure 3:**
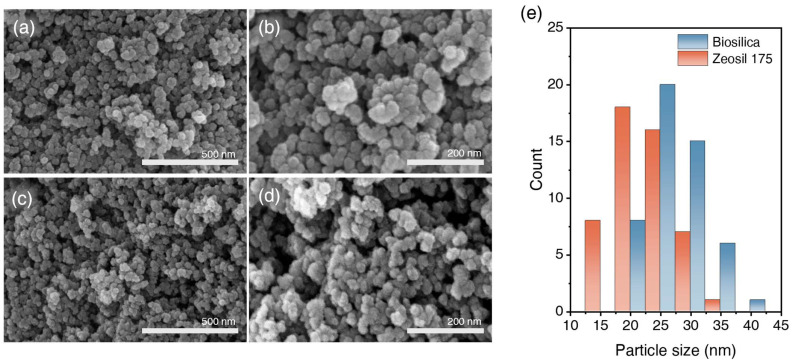
SEM micrographs of biosilica and Zeosil 175 at 100 k (100,000×) and 200 k (200,000×) magnifications, with (**a**) showing biosilica at 100 k, (**b**) biosilica at 200 k, (**c**) Zeosil 175 at 100 k, and (**d**) Zeosil 175 at 200 k. (**e**) Histogram showing the particle size distribution of biosilica and Zeosil 175, measured using ImageJ software (v1.53k).

The SEM micrographs of biosilica and Zeosil 175 are presented in Figure 3a–d. Both samples exhibit comparable morphological characteristics, featuring irregular, clustered aggregates typical of precipitated silica structures. Figure 3e shows the histogram of the primary particle sizes for biosilica and Zeosil 175. Zeosil 175 displays a primary particle size distribution ranging from 12.5 to 35 nm, while biosilica shows a distribution between 20 and 45 nm. The average primary particle size for Zeosil 175 is 19.89 nm, compared to 29.54 nm for biosilica, indicating that biosilica possesses a relatively larger primary particle size than Zeosil 175. Given the significant influence of primary particle size on aggregate formation under similar hydrophilicity conditions, biosilica demonstrates more pronounced agglomeration [39].

#### 3.1.5. Thermogravimetric Analysis

**Figure 4:**
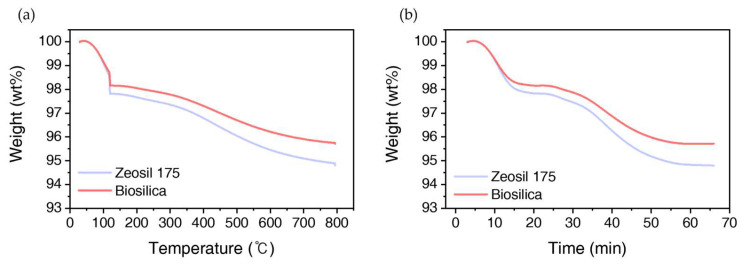
TGA curves of biosilica and Zeosil 175: (**a**) TGA curves showing weight percentage (wt%) as a function of temperature, and (**b**) TGA profiles with time as the independent variable.

The TGA curves of biosilica and Zeosil 175 are presented in Figure 4. Figure 4a displays the TGA curve with weight percentage plotted against temperature, while Figure 4b shows the TGA curve with time as the independent variable. Presenting the data in both formats allows for specialized profiles to quantify the silanol groups on the surface of each nanosilica, as described in the experimental section. The OH surface density of silica can be calculated using Equation (5) [26,27].

(5) #OH/nm2=α(#OHnm2)T2 × Specific Surface Area × wtT2+wtT1−wtT2MWH2ONA×2Specific Surface Area × wtT1

Here, T_2_ is 800 °C, and T_1_ is 120 °C, ${\mathrm{wt}}_{\mathrm{Ti}}$ represents the sample weight at the corresponding temperature Ti, ${\mathrm{MW}}_{H_{2}O}$ denotes the molecular weight of water, *N*_A_ is Avogadro’s constant, and α (=0.625) is a calibration factor. For silica, 1 OH/nm^2^ remains on the surface at T_2_ = 800 °C [40]. In Figure 4a, the weight loss observed around 100–120 °C is attributed to the evaporation of physically adsorbed water from the silica surface. The reduction in weight within this temperature range corresponds to humidity loss and is thus considered negligible. After maintaining the sample at 120 °C for 10 min to ensure complete water removal, this weight is defined as the baseline mass. From 120 to 800 °C, the silanol groups present on the silica surface are eliminated [26]. Silanol groups begin to condense and undergo dehydration above 170 °C, evolving water. Around 400 °C, less than half of the hydroxyl groups remain, and by the time the temperature reaches 750 °C, only 1.3 OH/nm^2^ of free, unpaired SiOH groups remain. Ultimately, at 800 °C, the concentration of hydroxyl groups is reduced to 1 OH/nm^2^. Additionally, volatile organic materials introduced during the silica synthesis are removed. To confirm the removal of humidity and the elimination of silanol groups from the silica surface, an isothermal hold at 800 °C for 10 min was conducted, as shown in Figure 4b. The weight loss at 120 °C for 10 min was minimal for biosilica, while Zeosil 175 showed a slight decrease. During the 800 °C step, both biosilica and Zeosil 175 experienced negligible weight loss. The calculated OH surface density of biosilica is 7.07 OH/nm^2^, while that of Zeosil 175 is 9.45 OH/nm^2^, consistent with FT-IR results that indicate a higher OH content in Zeosil 175. The thermal degradation analysis further confirms the stronger hydrogen bonding interactions in Zeosil 175-filled composites, although such interactions may also contribute to filler agglomeration at higher loadings. A critical factor in reinforcing rubber is the strength of filler–filer interactions; however, various other factors—such as crosslinking density, filler particle size, dispersion, surface chemistry, and specific surface area—also significantly influence the material’s properties [41]. Therefore, a multifaceted approach that examines these interrelated factors is essential rather than focusing on a single variable in isolation.

#### 3.1.6. The Yield of Basilica and Composition of Biosilica and Zeosil 175

The biosilica yield calculated via Equation (1) was about 89% (±4%). The chemical composition of biosilica and Zeosil 175 is shown in Table 2. The purity of biosilica was 96.4%, and that of Zeosil 175 was 95.5%, showing nearly similar values, indicating that biosilica possesses a purity comparable to that of Zeosil 175.

### 3.2. Results of Silicone Rubber Filled with Silica

#### 3.2.1. FT-IR

**Figure 5:**
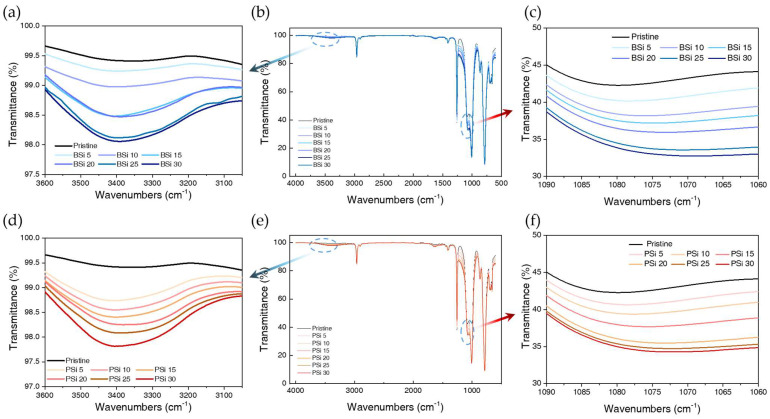
FT-IR spectra of pristine silicone rubber (0 phr) and composites filled with biosilica (**a**–**c**) and Zeosil 175 (**d**–**f**) at various loading levels (5, 10, 15, 20, 25 and 30 phr): (**a**,**d**) OH-stretching region (3600–3100 cm^−1^), (**b**,**e**) full spectra (4000–600 cm^−1^), and (**c**,**f**) Si-O-Si stretching region (1090–1060 cm^−1^).

The FT-IR spectra of silicone rubber filled with biosilica and Zeosil 175 are presented in Figure 5. Figure 5a shows the peak of silicone rubber incorporated with biosilica in the range of 3600–3050 cm^−1^. The intensity of the peak at 3450–3400 cm^−1^ increases with the increasing filler content in silicone rubber, indicating O–H asymmetrical stretching vibrations. This increase is attributed to the abundance of hydroxyl groups on the surface of biosilica, as illustrated in Figure 2b. Between 5 and 15 phr, the intensity of this peak shows a consistent upward trend; however, at 15, 20, 25, and 30 phr, the peak intensities become similar. The similarity in these peaks may be due to poor dispersion of biosilica. Figure 5c highlights the peak range of 1090–1060 cm^−1^, where the center of the peak for pristine silicone rubber is located at 1055–1050 cm^−1^. As the amount of silica added increases, the intensity of this peak strengthens, accompanied by blue shifting and broadening. This shifting and broadening are attributed to physisorption through hydrogen bonding between the hydroxyl groups of silica and the rubber matrix [42]. Figure 5d presents the peak for silicone rubber filled with Zeosil 175 in the 3600–3050 cm^−1^ range, while Figure 5f shows the peak range of 1090–1060 cm^−1^. In the case of PSi, the intensity of the peak around 3450–3400 cm^−1^ steadily increases with increasing silica content. Similarly, the peak range around 1090–1060 cm^−1^, as observed for BSi, also exhibits increased intensity with rising silica content, along with blue shifting and broadening due to physisorption.

#### 3.2.2. Thermogravimetric Analysis of PSi and BSi

**Figure 6:**
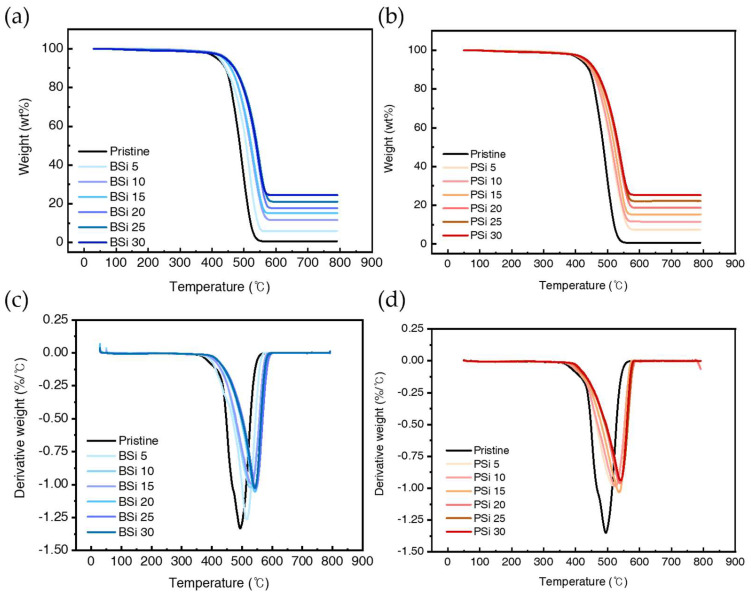
TGA and DTG curves of pristine and modified samples: (**a**) TGA curves of BSi samples with different concentrations (0, 5, 10, 15, 20, 25 and 30 phr), (**c**) corresponding DTG curves of BSi samples, (**b**) TGA curves of PSi samples with different concentrations (0, 5, 10, 15, 20, 25 and 30 phr), and (**d**) corresponding DTG curves of PSi samples.

The TGA and DTGA curves obtained from BSi and PSi with varying silica loading amounts are illustrated in Figure 6. The weight loss of pristine rubber approaches zero as the temperature increases. As shown in Figure 6c,d, the decomposition peak, referred to as the decomposition temperature (T_max_), is observed at 495 °C for pristine rubber. The T_max_ of silicone rubber filled with BSi and PSi increases with the loading level of silica, shifting to higher temperatures. However, at relatively high loading levels of silica, the degree of T_max_ shift becomes less pronounced. This result, regardless of the type, indicates that the thermal stability of silicone rubber is enhanced by the formation of a protective silica layer on the surface, which mitigates thermal penetration [43].

#### 3.2.3. Morphology of Silicone Rubber

**Figure 7:**
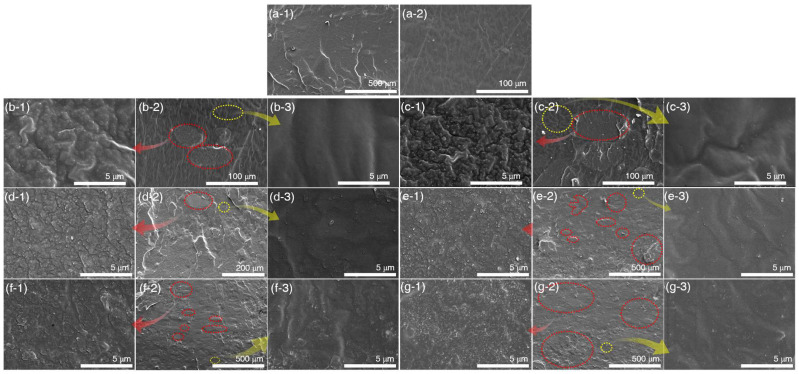
SEM micrographs of fractured silicone rubber surfaces: (**a**) pristine; (**b**) BSi 5; (**c**) BSi 10; (**d**) BSi 15; (**e**) BSi 20; (**f**) BSi 25; and (**g**) BSi 30. Excluding the pristine sample (**a**), images of samples (**b**–**g**) labeled with ‘**-1**’ represent high-magnification views of filler agglomeration areas marked with red circles, ‘**-2**’ denotes overview images showing the general morphological characteristics, and ‘**-3**’ presents detailed insets of uniformly dispersed regions marked with yellow circles.

**Figure 8:**
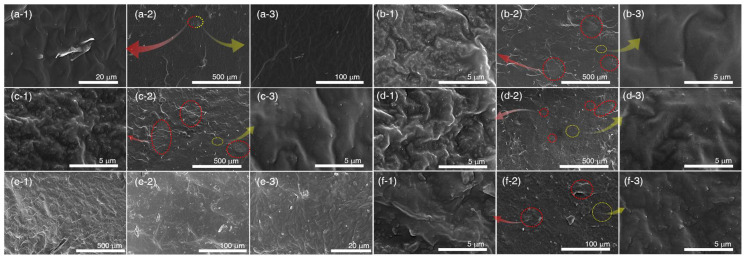
SEM micrographs of fractured silicone rubber surfaces: (**a**) PSi 5; (**b**) PSi 10; (**c**) PSi 15; (**d**) PSi 20; (**e**) PSi 25; and (**f**) PSi 30. Excluding the BSi 25 sample (**e**), images of samples (**a**–**f**) labeled with ‘**-1**’ represent high-magnification views of filler agglomeration areas marked with red circles, ‘**-2**’ denotes overview images showing the general morphological characteristics, and ‘**-3**’ presents detailed insets of uniformly dispersed regions marked with yellow circles. The images transition (**e-1**–**e-3**), with each subsequent image presenting a progressively higher magnification.

SEM micrographs of the fracture surfaces of pristine silicone rubber, BSi, and PSi are displayed in Figure 7 and Figure 8. On the surfaces of BSi 5 and BSi 10, as shown in Figure 7(b-2,c-2), agglomeration of biosilica, indicated by the red circles, is distinguishable from the non-filled silica regions marked by yellow circles, with sizes smaller than 100 μm. In comparison to the yellow circles, the biosilica particles and aggregates are embedded within the rubber of BSi 5 and BSi 10 (Figure 7(b-1,c-1)). For BSi 15 and above, as shown in Figure 7(d-2,e-2,f-2,g-2), the number of observed agglomerates increases, with sizes exceeding 100 μm. These agglomerates display a variety of sizes and begin to appear as a whole rather than in localized regions. The white areas inside the red circles, which are typically circular yet exhibit varied shapes as the silica content increases, represent the agglomeration of biosilica. The magnified regions in Figure 7(d-1,e-1,f-1,g-1) highlight the areas within the red circles that exhibit biosilica agglomeration. When compared to the SEM micrographs of non-agglomerated regions marked by yellow circles in Figure 7(d-3,e-3,f-3,g-3), a significantly greater presence of biosilica particles or aggregates is observed in the highlighted areas.

The observed degree of biosilica agglomeration with increasing filler content is attributed to the combined effects of particle size, specific surface area, and volume fraction at higher loadings. Figure 8 displays the SEM micrographs of the fractured surface of PSi. At low silica loading levels, such as PSi 5 to PSi 15, agglomerates of silica are detected locally. However, the distinction between agglomerates and the regions where silica particles cluster becomes less evident as the silica loading increases. Additionally, silica particles embedded in the surfaces of agglomerates at PSi 5 to PSi 20 show distinct differentiation from the surfaces in the yellow-highlighted regions. However, at PSi 25 and PSi 30, this distinction diminishes, as the silica agglomerates do not exhibit a significant increase in size. This phenomenon contrasts with the behavior observed in BSi, indicating that dispersion of silica is more effective in PSi composites.

The primary particle size of Zeosil 175 is 19.89 nm, whereas that of biosilica is 29.54 nm. Due to the larger particle size of biosilica, the aggregates formed by bound rubber are correspondingly larger, leading to agglomerates that are greater in size compared to those formed by Zeosil 175 [39]. At low volume fractions, the absolute amount of silicone gum is higher relative to the filler content, and the limited quantity of fillers restricts sufficient shear application during the milling process, preventing disruption of silica agglomerate formation. Consequently, both PSi and BSi exhibit agglomeration under these conditions. As the volume fraction of the filler increases, Zeosil 175, with its relatively lower specific surface area, forms agglomerates that are more easily broken down during milling, resulting in a more uniform dispersion within the silicone matrix. In contrast, biosilica, with its larger specific surface area, experiences stronger filler–filer interactions, resulting in more pronounced agglomeration. During milling, even if these agglomerates are partially broken, the larger agglomerates tend to persist in certain areas, leading to a more localized distribution of large agglomerates [44]. This ultimately causes BSi to exhibit poorer dispersion compared to PSi despite undergoing the same milling process.

#### 3.2.4. Chemo-Mechanical Properties

##### Tensile Strength

**Figure 9:**
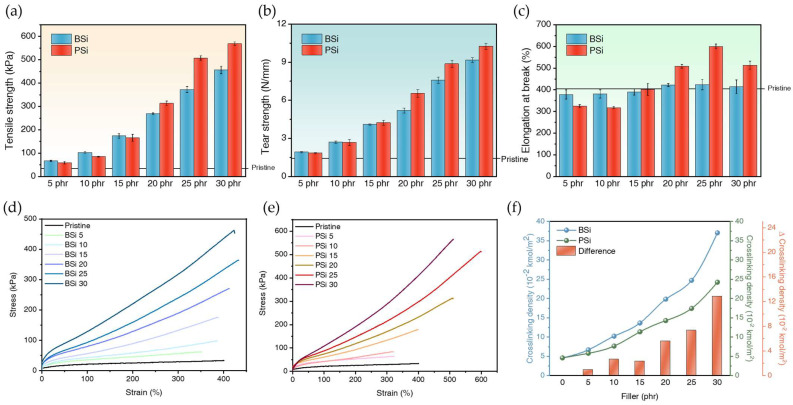
Mechanical properties of BSi and PSi at various filler loadings, showing (**a**) tensile strength, (**b**) tear strength, and (**c**) elongation at the break. Stress–strain curves of silicone composites containing (**d**) BSi and (**e**) PSi. (**f**) Crosslinking density as a function of filler content for BSi- and PSi-filled composites.

In Figure 9a, which presents the tensile strength results of BSi and PSi, it is observed that BSi exhibits comparable tensile strength to PSi in the range of 5–15 phr. However, at filler loadings above 20 phr, PSi demonstrates superior tensile strength. Additionally, when examining the strain-stress curves in Figure 9d,e, it can be observed that at 5–15 phr, the tensile strength values of BSi and PSi are nearly identical or BSi is slightly higher, whereas at loadings above 20 phr, PSi exhibits superior values. Detailed tensile strength values are provided in Table 3. The observed behavior can be attributed to the lack of significant differences in dispersion between Zeosil 175 and biosilica at low silica loadings. At these lower loadings, biosilica, due to its higher specific surface area, fosters more extensive filler–matrix interactions. Despite Zeosil 175 having a greater quantity of hydroxyl groups on its surface, the tensile strength of biosilica-filled composites remains comparable or slightly higher, indicating that rubber reinforcement is influenced not only by OH content but also by the specific surface area of the filler. This emphasizes the importance of surface area alongside chemical content in the rubber reinforcement process. Conversely, the reduction in tensile strength at filler loadings above 20 phr can be attributed to the poor dispersion of biosilica within the matrix, as observed in the SEM micrographs. When silica nanoparticles are evenly dispersed in the rubber, they form only small aggregates, leading to stable filler–matrix interactions and effective reinforcement throughout the rubber matrix. However, when poor dispersion causes agglomeration, larger aggregates form, resulting in filler–filer interactions dominating over filler–matrix interactions. Consequently, the load during deformation is not uniformly transmitted across the filler, causing stress concentration at the agglomerates. This leads to the breakdown of the silica network and the formation of micro-cracks, ultimately reducing the increase in tensile strength.

##### Modulus at 100% and 300% Elongation

In Table 3, the values for E100 and E300 indicate that at lower filler loadings, the values for BSi and PSi are nearly identical or slightly favor PSi. While BSi exhibits higher values at higher loadings, the tensile strength and elongation are greater for PSi near higher filler loadings. This can be explained by the fact that, at the same silica loading, BSi experiences higher stress at relatively lower elongation values compared to PSi due to the presence of agglomerates. Consequently, BSi experiences breakage at lower elongation than PSi, as the agglomerates concentrate stress in localized regions, leading to earlier fracture.

##### Tear Strength

Figure 9b presents a graph displaying the tear strength of BSi and PSi. Similar to the tensile properties, the tear strength exhibits a comparable trend, with BSi showing greater tear strength at low silica loadings (5–10 phr). However, at 15 phr, PSi either surpasses or matches the tear strength levels of BSi, resulting in an intersection where both materials exhibit nearly identical properties. At silica loadings above 20 phr, PSi demonstrates superior tear strength compared to BSi. This finding aligns with the explanation provided for the tensile property behavior: at low silica loadings, the higher specific surface area of biosilica enhances reinforcement, while at higher loadings, increased agglomeration in biosilica leads to reduced reinforcement due to the Payne effect. Consequently, PSi, characterized by more uniform dispersion, outperforms BSi in tear strength at higher loadings.

##### Elongation at Break

The elongation at break values for BSi and PSi are presented in Figure 9c and Table 3. Additionally, as shown in Figure 9d,e, BSi 5, BSi 10, PSi 5, and PSi 10 exhibit lower elongation values compared to pristine silicone rubber. This reduction in elongation is attributed to the incorporation of silica, which is less elastic than pure silicone gum. At low filler loadings, the reinforcement effect is less pronounced, resulting in decreased material elasticity and lower elongation at the break. However, as the silica content exceeds 15 phr, elongation increases compared to pristine rubber. For BSi, elongation slightly increases but decreases after 25 phr due to the formation of agglomerates at high silica content, which induces micro-cracks. This condition increases stress tolerance but reduces elongation, ultimately resulting in a stiffer material. In the case of PSi, elongation continues to increase up to 25 phr, after which it decreases to 30 phr, suggesting that the dispersion of Zeosil 175 at 30 phr is less effective in terms of elongation than at lower filler loadings, as can also be seen in Figure 9d.

#### 3.2.5. Crosslinking Density

The crosslinking density of silicone rubber was measured using the solvent-swelling method, with toluene as the solvent, and the data were analyzed using the Flory-Rehner equation. As shown in Figure 9f, the crosslinking density of BSi is slightly higher than that of PSi at filler loadings below 15 phr. This difference can be attributed to the higher specific surface area of biosilica, which enhances filler–matrix interactions, and the presence of more agglomerates in BSi at lower silica loadings compared to PSi, even when dispersion is adequate. At filler loadings above 20 phr, the difference in crosslinking density between BSi and PSi increases, which can be explained by the agglomeration of biosilica that leads to heightened filler–filer interactions. Specifically, the hydrogen bonding among biosilica particles within the agglomerates contributes to this observed difference [45]. As the crosslinking density increases, both BSi and PSi exhibit improved tensile strength. The sudden increase in crosslinking density at 30 phr coincides with the significant formation of the Payne effect, indicating that the samples become stiffer. Increased stiffness allows the material to endure higher stress at the same strain, but it also restricts the sample from reaching higher strain values. As a result, the elongation at the break of PSi at 30 phr decreases. Additionally, in the case of BSi, the agglomeration of biosilica leads to higher E100 and E300 values, and tensile strength increases with biosilica loading. However, it is evident that the elongation at the break does not improve, even with low biosilica loading.

#### 3.2.6. Analysis of Cured Silicone Rubber

**Figure 10:**
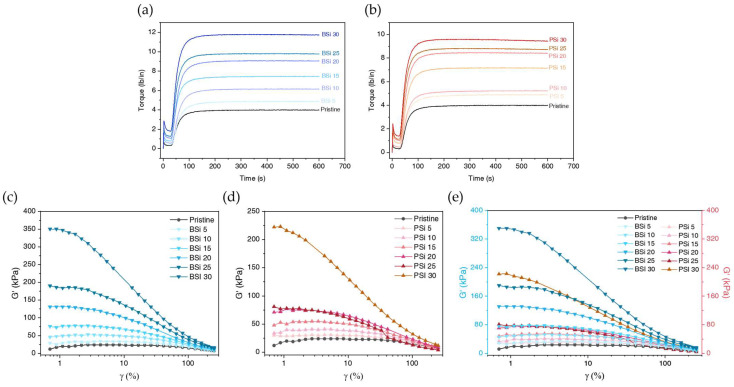
Torque evolution during vulcanization for silicone composites with biosilica (**a**) and precipitated silica (**b**) at various loadings (5–30 phr). Strain-dependent storage modulus (G′) of BSi (**c**) and PSi (**d**) filled composites, respectively, exhibiting the Payne effect due to filler network breakdown at higher strains, and (**e**) Comparative strain sweep of BSi and PSi composites.

The rheological characteristics of cured silicone rubber are illustrated in Table 4, while the curing curves of BSi and PSi at various silica loading levels, including pristine silicone rubber, are shown in Figure 10a,b. The ts2 for both BSi and PSi decreases with increasing silica loading levels, with minimal differences observed between the two types of silica. This suggests that the type of silica does not significantly influence the onset of crosslinking [46]. The tc90 values for both types of silica, compared to pristine rubber, increase. This is due to filler–rubber interactions, as silica particles interact with the silicone rubber matrix, forming a bound rubber layer around the filler particles. This bound rubber restricts the mobility of polymer chains, slowing down the curing process. The tc90 decreases as silica loading increases, with BSi consistently exhibiting slightly higher values. This behavior can be attributed to the larger specific surface area of biosilica, which causes more disruption during the vulcanization process, leading to a delay in the crosslinking reaction [21]. The reason for the decrease in ts2 and tc90 as silica loading increases is that, as silica loading increases, the heat generation during the vulcanization test increases due to the die’s oscillation. The torque difference (M_H_-M_L_) serves as a measure of the dynamic shear modulus, indirectly indicating the extent of crosslinking density within the composites [47,48]. The torque differences of BSi and PSi, presented in Table 4, are consistent with the crosslinking density obtained via the solvent-swelling method, as shown in Figure 9f. Thus, the findings confirm that the slightly higher torque difference for BSi is due to the filler–rubber interaction of biosilica, which is influenced by its high specific surface area at low silica loadings, along with the elevated crosslink density resulting from hydrogen bonding in the agglomerated biosilica at high silica loadings.

#### 3.2.7. Viscoelastic Characteristics of Uncured Silicone Compound

The dynamic strain amplitude sweep results for the storage modulus are presented in Figure 10c–e. The Payne effect, as defined by Payne, refers to the difference between the estimated shear modulus (G_0_) at 0% oscillation strain and the shear modulus (G_∞_) at infinite strain [48]. The Payne effect is associated with the breakdown of the filler network and the entrapment of rubber within it, caused by shear forces as the oscillation amplitude increases [49]. In Figure 10c,d, the storage modulus (G’_0_) of BSi and PSi, both filled with silica, rises with increasing silica loading levels. However, as shown in Figure 10e, the Payne effect of BSi is greater than that of PSi at the same silica loading levels. Furthermore, with the exception of PSi at 30 phr, all PSi samples are less strain-dependent compared to BSi at 20 phr. This indicates that the filler network of biosilica is more developed than that of Zeosil 175 below 30 phr, including at 25 phr, suggesting that the agglomeration of biosilica is more pronounced than that of Zeosil 175. The increasing gap in the Payne effect at PSi 30 is significantly larger than the trend observed in lower PSi samples, indicating that agglomeration in PSi 30 becomes more pronounced compared to samples below 25 phr. This result aligns well with Figure 9c, where PSi 30 exhibits increased stiffness relative to the other PSi samples.

## 4. Conclusions

Biosilica derived from rice husk was synthesized using an environmentally friendly method. Both biosilica and conventional silica (Zeosil 175) were characterized using various analytical techniques, and silicone rubber filled with different amounts of each type of silica was compared using chemo-mechanical methods. The nitrogen adsorption–desorption isotherms revealed that the specific surface area of biosilica (159.52 m^2^/g) is higher than that of Zeosil 175 (144.90 m^2^/g). FT-IR and TGA analyses indicated that Zeosil 175 (9.45 OH/nm^2^) has a greater number of hydroxyl groups on its surface compared to biosilica (7.07 OH/nm^2^). The primary particle size of biosilica (29.54 nm) is larger than that of Zeosil 175 (19.89 nm), as determined from SEM micrographs and particle size distribution analysis. In TGA and DTGA curves, biosilica and Zeosil 175 contributed to thermal protection for silicone rubber filled with these silica nanoparticles. The dispersion of biosilica and Zeosil 175 was characterized through SEM micrographs. At low silica content (5–15 phr), both biosilica and Zeosil 175 exhibit similar dispersion due to a lack of shear stress from the silicone gum. However, at a high silica content (20–30 phr), agglomeration of biosilica is more pronounced than that of Zeosil 175 due to its higher specific surface area, which reinforces filler–filer interactions and leads to larger primary particle sizes despite the relatively lower hydroxyl content on its surface. Tensile strength results indicate that, at lower silica loadings, biosilica exhibits comparable reinforcing performance to Zeosil 175, which may be due to its higher specific surface area. Conversely, at higher silica loadings, the reinforcing effect of biosilica diminishes compared to Zeosil 175 due to poor dispersion. The crosslinking density and torque difference in the curing curve for BSi are greater than those for PSi, and their increase becomes more pronounced at higher loading levels due to hydrogen bonding within agglomerates. The significant agglomeration and poor dispersion are corroborated by the dynamic strain amplitude sweep results. The Payne effect of BSi is greater than that of PSi, and for all PSi samples except for PSi 30, the initial storage modulus at 0% strain is lower than that of BSi at 20 phr. This study is expected to provide a foundation for utilizing rice husk-derived biosilica or other biomaterial-derived silicas as substitutes for conventional silica, not only for reinforcing rubber but also for other applications.

## Figures and Tables

**Table 1:** Compositions of silicone rubber filled with biosilica (BSi) or Zeosil 175 (PSi).

	BSi						PSi					
phr	5	10	15	20	25	30	5	10	15	20	25	30
PDMS gum	100	100	100	100	100	100	100	100	100	100	100	100
Curative	1	1	1	1	1	1	1	1	1	1	1	1
Biosilica	5	10	15	20	25	30						
Zeosil 175							5	10	15	20	25	30

**Table 2:** The chemical composition of the biosilica obtained from the rice husk and Zeosil 175.

Compound	Biosilica	Zeosil 175
SiO_2_	96.4	95.5
Na_2_O	2.15	2.6
SO_3_	0.683	0.704
Al_2_O_3_	0.639	1.01
CaO	0.0396	0.0237
MgO	0.0384	0.0857
SnO_2_	0.0333	0.0283
K_2_O	0.0205	-
P_2_O_5_	0.0055	-
Fe_2_O_3_	0.0045	0.0081
ZnO	0.0019	-
Cl	-	0.0244
ZrO_2_	-	0.0014
SnO_2_	-	0.0283

**Table 3:** Modulus at 100% elongation and 300% elongation, tensile strength, elongation at the break of pristine silicone rubber, silicone rubber filled with biosilica and Zeosil 175.

Sample	Modulus E100 (kPa)	Modulus E300 (kPa)	Tensile Strength (kPa)	Tear Strength	Elongation at Break (%)
Pristine	21.38 (±1.56)	29.32 (±1.916)	33.73 (±1.257)	1.49 (±0.0349)	400.40 (±13.72)
BSi 5	35.99 (±4.119)	56.29 (±2.157)	65.44 (±2.893)	1.91 (±0.049)	351.40 (±21.38)
BSi 10	43.25 (±1.177)	77.57 (±7.159)	101.73 (±3.6285)	2.7 (±0.0981)	386.10) (±19.39)
BSi 15	56.39 (±4.021)	133.08 (±5.492)	172.67 (±10.5912)	4.09 (±0.049)	388.10 (±14.805)
BSi 20	79.73 (±4.021)	189.46 (±10.983)	267.46 (±3.7265)	5.2 (±0.1961)	412.10 (±7.135)
BSi 25	93.07 (±12.945)	239.87 (±27.85)	371.38 (±12.5525)	7.58 (±0.2452)	433.25 (±23.85)
BSi 30	129.06 (±7.159)	317.93 (±13.337)	455.26 (±15.9358)	9.15 (±0.1961)	424.20 (±31.825)
PSi 5	42.95 (±4.217)	68.76 (±6.178)	57.61 (±5.5408)	1.83 (±0.049)	323.05 (±7.335)
PSi 10	41.68 (±5.001)	80.51 (±4.609)	83.94 (±1.8142)	2.67 (±0.2452)	319.80 (±5.12)
PSi 15	61.19 (±11.964)	133.4 (±21.084)	164.26 (±15.0042)	4.22 (±0.1961)	398.95 (±28.03)
PSi 20	74.33 (±5.394)	170.24 (±2.255)	312.51 (±9.4634)	6.54 (±0.2942)	510.35 (±8.435)
PSi 25	87.77 (±5.59)	214.77 (±10.003)	506.58 (±9.6596)	8.86 (±0.2942)	599.55 (±11.185)
PSi 30	108.07 (±23.241)	288.22 (±15.494)	567.90 (±7.4040)	10.25 (±0.2452)	510.70 (± 8.955)

**Table 4:** The scorch time (ts2), tc90, minimum torque (M_L_), maximum torque (M_H_), and Δ torque between M_H_ and M_L_ of vulcanized silicone rubber filled with biosilica and Zeosil 175.

Sample	ts2 (s)	tc90 (s)	M_L_ (lb/in)	M_H_ (lb/in)	M_H_-M_L_ (lb/in)
Pristine	54	97	0.31	4.01	3.7
BSi 5	54	109	0.41	4.9	4.49
BSi 10	49	104	0.56	6.18	5.62
BSi 15	42	90	0.79	7.46	6.67
BSi 20	43	96	1.07	9.09	8.01
BSi 25	40	85	1.27	9.82	8.54
BSi 30	39	84	1.81	11.8	9.99
PSi 5	51	104	0.43	4.91	4.47
PSi 10	49	99	0.45	5.24	4.79
PSi 15	45	93	0.78	7.18	6.4
PSi 20	43	90	1.01	8.46	7.46
PSi 25	40	82	1.11	8.82	7.71
PSi 30	39	81	1.38	9.6	8.21

## Data Availability

Data is contained within the article.

## References

[1] Satbaev B., Yefremova S., Zharmenov A., Kablanbekov A., Yermishin S., Shalabaev N., Satbaev A., Khen V. (2021). Rice Husk Research: From Environmental Pollutant to a Promising Source of Organo-Mineral Raw Materials. Materials.

[2] Ghosh R. (2013). A Review Study on Precipitated Silica and Activated Carbon from Rice Husk. J. Chem. Eng. Process Technol..

[3] Sharma N.K., Williams W.S., Zangvil A. (1984). Formation and Structure of Silicon Carbide Whiskers from Rice Hulls. J. Am. Ceram. Soc..

[4] Bakar R.A., Yahya R., Gan S.N. (2016). Production of High Purity Amorphous Silica from Rice Husk. Procedia Chem..

[5] Fernandes I.J., Calheiro D., Sánchez F.A.L., Camacho A.L.D., Rocha T.L.A.d.C., Moraes C.A.M., Sousa V.C.d. (2017). Characterization of Silica Produced from Rice Husk Ash: Comparison of Purification and Processing Methods. Mater. Res..

[6] Ma X., Zhou B., Gao W., Qu Y., Wang L., Wang Z., Zhu Y. (2012). A Recyclable Method for Production of Pure Silica from Rice Hull Ash. Powder Technol..

[7] Riveros H., Garza C. (1986). Rice Husks as a Source of High Purity Silica. J. Cryst. Growth.

[8] Liou T.-H., Yang C.-C. (2011). Synthesis and Surface Characteristics of Nanosilica Produced from Alkali-Extracted Rice Husk Ash. Mater. Sci. Eng. B.

[9] Yalçin N., Sevinç V. (2001). Studies on Silica Obtained from Rice Husk. Ceram. Int..

[10] Liu Z., Hu D., Zheng C., Yu K., Zhang X., Ma W. (2024). Bioinspired High-Performance Silicone Elastomers by Catalyst-Free Dopamine Cross-Linking. Ind. Eng. Chem. Res..

[11] Eduok U., Faye O., Szpunar J. (2017). Recent Developments and Applications of Protective Silicone Coatings: A Review of PDMS Functional Materials. Prog. Org. Coat..

[12] Mirzadeh H., Khorasani M.T. (2003). Physical, Mechanical, and Biocompatibility Evaluation of Three Different Types of Silicone Rubber. J. Appl. Polym. Sci..

[13] Yilgör E., Yilgör I. (2014). Silicone Containing Copolymers: Synthesis, Properties and Applications. Prog. Polym. Sci..

[14] Song J.S., Lee S., Cha G.C., Jung S.H., Choi S.Y., Kim K.H., Mun M.S. (2005). Surface Modification of Silicone Rubber by Ion Beam Assisted Deposition (IBAD) for Improved Biocompatibility. J. Appl. Polym. Sci..

[15] Shit S.C., Shah P. (2013). A Review on Silicone Rubber. Natl. Acad. Sci. Lett..

[16] Mazurek P., Vudayagiri S., Ladegaard Skov A. (2019). How to Tailor Flexible Silicone Elastomers with Mechanical Integrity: A Tutorial Review. Chem. Soc. Rev..

[17] Cochrane H., Lin C.S. (1993). The Influence of Fumed Silica Properties on the Processing, Curing, and Reinforcement Properties of Silicone Rubber. Rubber Chem. Technol..

[18] Boonstra B.B., Cochrane H., Dánnenberg E.M. (1975). Reinforcement of Silicone Rubber by Particulate Silica. Rubber Chem. Technol..

[19] Gomes C.M., Cheung N., Gomes G.M., Sousa A.K., Peruzzi A.P. (2021). Improvement of Water Resistance in Magnesia Cements with Renewable Source Silica. Constr. Build. Mater..

[20] Eissa M.M., Botros S.H., Diab M., Shafik E.S., Rozik N.N. (2023). Rice Husk Fibers and Their Extracted Silica as Promising Bio-Based Fillers for EPDM/NBR Rubber Blend Vulcanizates. Clean Technol. Environ. Policy.

[21] Choophun N., Chaiammart N., Sukthavon K., Veranitisagul C., Laobuthee A., Watthanaphanit A., Panomsuwan G. (2022). Natural Rubber Composites Reinforced with Green Silica from Rice Husk: Effect of Filler Loading on Mechanical Properties. J. Compos. Sci..

[22] Sethuramalingam V.C., Prabagaran S., Ganesan K. (2021). Studies on Influence of Silica Filler and Rice Husk Ash on the Mechanical Properties of Vulcanized Hybrid Rubber Composite. Mater. Today Proc..

[23] Jiang Z., Fu Z., Ning K. Study on Properties of Precipitated and Fumed Silica Reinforced Polydimethylsiloxane Silicone Rubber. Proceedings of the 2023 IEEE 4th International Conference on Electrical Materials and Power Equipment (ICEMPE).

[24] Azmi M.A., Mahzan S., Ahmad S., Salleh S.M., Rahman H.A., Choiron M.A., Ismail A., Taib H. (2019). Vibration Exposure of Polydimethylsiloxane (PDMS) Reinforced Silica (SiO_2_): Comparison of Different Source of Silica (SiO_2_) as Filler. IOP Conf. Ser. Mater. Sci. Eng..

[25] Kalapathy U., Proctor A., Shultz J. (2000). A Simple Method for Production of Pure Silica from Rice Hull Ash. Bioresour. Technol..

[26] Mueller R., Kammler H.K., Wegner K., Pratsinis S.E. (2003). OH Surface Density of SiO_2_ and TiO_2_ by Thermogravimetric Analysis. Langmuir.

[27] Wisser F.M., Abele M., Gasthauer M., Müller K., Moszner N., Kickelbick G. (2012). Detection of Surface Silanol Groups on Pristine and Functionalized Silica Mixed Oxides and Zirconia. J. Colloid Interface Sci..

[28] (2016). Standard Test Methods for Vulcanized Rubber and Thermoplastic Elastomers—Tension.

[29] (2012). Standard Test Method for Tear Strength of Conventional Vulcanized Rubber and Thermoplastic Elastomers.

[30] Shim S.E., Isayev A.I. (2001). Ultrasonic Devulcanization of Precipitated Silica-Filled Silicone Rubber. Rubber Chem. Technol..

[31] Marzocca A.J., Rodríguez Garraza A.L., Mansilla M.A. (2010). Evaluation of the Polymer–Solvent Interaction Parameter *χ* for the System Cured Polybutadiene Rubber and Toluene. Polym. Test..

[32] Scott R.L., Magat M. (1945). The Thermodynamics of High-Polymer Solutions: I. The Free Energy of Mixing of Solvents and Polymers of Heterogeneous Distribution. J. Chem. Phys..

[33] Lu H., Feng S. (2017). Supramolecular Silicone Elastomers with Healable and Hydrophobic Properties Crosslinked by “Salt-Forming Vulcanization”. J. Polym. Sci. Part Polym. Chem..

[34] Fanse S., Bao Q., Zou Y., Wang Y., Burgess D.J. (2022). Impact of Polymer Crosslinking on Release Mechanisms from Long-Acting Levonorgestrel Intrauterine Systems. Int. J. Pharm..

[35] Jyoti A., Singh R.K., Kumar N., Aman A.K., Kar M. (2021). Synthesis and Properties of Amorphous Nanosilica from Rice Husk and Its Composites. Mater. Sci. Eng. B.

[36] Biswas R.K., Khan P., Mukherjee S., Mukhopadhyay A.K., Ghosh J., Muraleedharan K. (2018). Study of Short Range Structure of Amorphous Silica from PDF Using Ag Radiation in Laboratory XRD System, RAMAN and NEXAFS. J. Non-Cryst. Solids.

[37] Xu T., Jia Z., Luo Y., Jia D., Peng Z. (2015). Interfacial Interaction between the Epoxidized Natural Rubber and Silica in Natural Rubber/Silica Composites. Appl. Surf. Sci..

[38] Lee J.H., Kwon J.H., Lee J.-W., Lee H., Chang J.H., Sang B.-I. (2017). Preparation of High Purity Silica Originated from Rice Husks by Chemically Removing Metallic Impurities. J. Ind. Eng. Chem..

[39] Shui Y., Huang L., Wei C., Sun G., Chen J., Lu A., Sun L., Liu D. (2021). How the Silica Determines Properties of Filled Silicone Rubber by the Formation of Filler Networking and Bound Rubber. Compos. Sci. Technol..

[40] Curthoys G., Davydov V.Y., Kiselev A.V., Kiselev S.A., Kuznetsov B.V. (1974). Hydrogen Bonding in Adsorption on Silica. J. Colloid Interface Sci..

[41] Ansarifar A., Lim B. (2006). Reinforcement of Silicone Rubber with Precipitated Amorphous White Silica Nanofiller—Effect of Silica Aggregates on the Rubber Properties. J. Rubber Res..

[42] Kralevich M.L., Koenig J.L. (1998). FTIR Analysis of Silica-Filled Natural Rubber. Rubber Chem. Technol..

[43] Tarrío-Saavedra J., López-Beceiro J., Naya S., Artiaga R. (2008). Effect of Silica Content on Thermal Stability of Fumed Silica/Epoxy Composites. Polym. Degrad. Stab..

[44] Liu D., Song L., Song H., Chen J., Tian Q., Chen L., Sun L., Lu A., Huang C., Sun G. (2018). Correlation between Mechanical Properties and Microscopic Structures of an Optimized Silica Fraction in Silicone Rubber. Compos. Sci. Technol..

[45] Bernal-Ortega P., Anyszka R., Morishita Y., di Ronza R., Blume A. (2024). Determination of the Crosslink Density of Silica-Filled Styrene Butadiene Rubber Compounds by Different Analytical Methods. Polym. Bull..

[46] Tong Y., Liu H., Chen A., Guan H., Kong J., Liu S., He C. (2018). Effect of Surface Chemistry and Morphology of Silica on the Thermal and Mechanical Properties of Silicone Elastomers. J. Appl. Polym. Sci..

[47] Bendjaouahdou C., Bensaad S. (2011). Properties of Polypropylene/(Natural Rubber)/Organomontmorillonite Nanocomposites Prepared by Melt Blending. J. Vinyl Addit. Technol..

[48] Lolage M., Parida P., Gupta A., Rautaray D. (2022). Synergistic Effects of Silica and Nanoclay on Curing Characteristics, Processing Behaviour and Mechanical Properties of Solution Styrene Butadiene Rubber (SBR)–Based Tire Tread Compounds. Emergent Mater..

[49] Lipińska M., Soszka K. (2019). Viscoelastic Behavior, Curing and Reinforcement Mechanism of Various Silica and POSS Filled Methyl-Vinyl Polysiloxane MVQ Rubber. Silicon.

